# Distinct biochemical phenotypes of HIV exposed infants driven by antiviral medication

**DOI:** 10.64898/2026.01.28.26344948

**Published:** 2026-02-02

**Authors:** Shujian Zheng, Joshua M. Mitchell, Jasmine Chong, Jennifer Canniff, Michael J Johnson, Maheshwor Thapa, Elizabeth Aiken, Shabir Madhi, Adriana Weinberg, Shuzhao Li

**Affiliations:** 1 The Jackson Laboratory for Genomic Medicine, 10 Discovery Drive, Farmington, CT 06032, USA; 2 Department of Medicine, University of Colorado Anschutz Medical Campus, Aurora, Colorado, USA; 3 Department of Pediatrics, University of Colorado Anschutz Medical Campus, Aurora, Colorado, USA; 4 Department of Pathology, University of Colorado Anschutz Medical Campus, Aurora, Colorado, USA; 6 South African Medical Research Council Vaccines and Infectious Diseases Analytics Research Unit and Department of Science and Technology/National Research Foundation South African Research Chair Initiative in Vaccine Preventable Diseases, Faculty of Health Sciences, University of the Witwatersrand, Johannesburg, South Africa; 7 African Leadership in Vaccinology Expertise, Faculty of Health Sciences, University of the Witwatersrand, Johannesburg, South Africa; 8 University of Connecticut School of Medicine, Farmington, CT 06032, USA

## Abstract

Pregnant women with HIV control viral replication with antiretrovirals and give birth to HIV-exposed uninfected infants (HEU). The children, however, exhibit increased morbidity and mortality due to severe infections, as well as cognitive and growth abnormalities. In this study, we performed high-resolution, untargeted metabolomics on 123 HIV-exposed mother-baby pairs and 117 control pairs without HIV. High concentrations of the antiretroviral efavirenz and its metabolites were detected in maternal blood and cord blood. The metabolomic differences between HEU participants and controls reflect perturbed pathways of steroids, tryptophan and bile acids, and they largely consisted of metabolites that were correlated with efavirenz concentrations within the HEU group. The results suggest a major contribution of the drug to the abnormal biochemical profile of HEU infants born to mothers treated with efavirenz.

Epidemiological evidence shows that in utero exposures to infections and chemical substances, including therapeutics, have profound impacts on population health[1–5]. A well-studied area is children born to mothers with HIV, who are not infected by HIV (HIV-Exposed Uninfected or HEU). The HEU children are known to exhibit increased morbidity and mortality due to severe infections, as well as cognitive and growth abnormalities when compared to HIV-unexposed children (HIV-Unexposed Uninfected or HUU)[6–9]. This poses a significant health burden with over 16 million affected children in 2023, most of whom live in sub-Saharan Africa [9]. The extent to which exposure to antiretrovirals contributes to undesirable health outcomes is incompletely understood.

Untargeted metabolomics has recently become a promising approach by quantifying the exposures and biological effects simultaneously[10–13]. Previous metabolomic studies in HEU infants were technologically limited and measured small numbers of metabolites/lipids in small cohorts [14–17]. In this study, we performed untargeted, high-resolution metabolomics and lipidomics in 123 HEU mother-infant pairs and 117 HUU control pairs. The demographic characteristics of the study population was previously described[18]. Notably, all mothers with HIV received antiretroviral therapy during pregnancy, including efavirenz in 92% of them. The metabolomic analysis was performed with hydrophilic interaction chromatography (HILIC), and lipidomics reverse phase (RP) chromatography, both using positive and negative electrospray ionization (ESI+/−, see Methods). The data were generated on plasma samples from maternal peripheral blood collected at delivery and cord blood. Unsupervised principal component analysis (PCA) showed clear separations of the mothers and infants by both sampling method and by HIV status (Fig. 1a, Suppl. Fig. 1a). The difference was driven by large numbers of metabolites with significantly different concentrations (FDR < 0.05, 658 RP ESI-features Fig. 1b; 1827 HILIC ESI-features, Suppl. Fig. 1b).

HEU cord blood displayed altered steroid metabolism, which included lower pregnenolone, pregnenolone sulfate, estrone-3-sulfate, but higher cholesterol derivatives (Fig. 1c). Notably, many sulfated metabolites were significant in the result, possibly due to the involvement of sulfotransferases in drug metabolism [19]. Disruptions of sugar and bilirubin metabolism were also detected (Fig. 1d,e; Suppl Fig. 2a). Tryptophan metabolites, such as serotonin sulfate and doptamine 3-O-sulfate, show different direction of alteration in HEU and HUU infants (Suppl Fig. 2b). Differentially abundant bile acids include chenodeoxycholic acid and derivatives (Suppl Fig. 2c). Multiple polyunsaturated fatty acids (PUFAs), commonly linked to CYP2B6 activity, were perturbed (Suppl Fig. 2d). Given the critical roles of pregnenolone and PUFAs in fetal development[20, 21], this provides a potential mechanism for impaired growth in HEU infants. The dysregulation of bile acids and tryptophan metabolisms may be responsible for their issues in immune and neural developments[22–24].

Differences in steroids and estrogens were more significant in the mothers than infants, including reductions in DHEA-S, estrone sulfate, glucuronidated cholesterol and sulfated steroids (Suppl. Fig. 2e,f). Metabolites with significantly different concentrations in HEU and HUU were shared between mothers and infants. Additional examples included higher bilirubin derivatives in HUU, elevated taurocholic acid in HEU and decreased PAGln (Suppl. Fig. 2g). Network analysis using the mummichog software[25] highlighted activities in steroid metabolism in both HEU mothers and infants (Suppl. Fig. 3).

Among the top differential features by HEU status were efavirenz and its metabolites (Fig. 2a). The chemical identity of efavirenz and its metabolite 8-hydroxyefavirenz was confirmed by matched mass-to-charge ratio (m/z), retention time and MS/MS spectra to authentic standards (Fig. 2b). The drug was detected in the cord blood of most HEU infants, and strong correlations were observed between efavirenz and 8-hydroxyefavirenz (Fig. 2c). Similar patterns were observed in mothers with HIV (Fig. 2d,e; Suppl. Fig. 4a). These data are consistent with previous studies demonstrating that the drug readily crosses the placenta and exposes the fetuses at high doses[26, 27].

To further understand the role of efavirenz in the metabolic alterations of HEU mother-infant pairs, we carried out a metabolome-wide association study (MWAS), taking advantage of the broad range of efavirenz concentrations in HEU infants and their mothers. In the HEU infants, 483 features were significantly correlated with efavirenz (Pearson’s correlation, FDR < 0.01), as visualized on a Manhattan plot along LC elution time (Fig. 2f, top). Compared to the features significantly different between HEU and HUU (Fig. 2f, bottom), the major feature clusters appeared strikingly similar. The similarity extended to the maternal data (Suppl Fig. 4b). Indeed, comparisons between the four groups, efavirenz MWAS and HEU/HUU differences in infants and mothers, show that the significant features largely overlapped (Fig. 2g). The top pathways enriched in efavirenz-correlated metabolites also differentiated participants by HIV status (Fig. 2h).

In summary, drastic metabolic alterations were observed in HEU infants and their mothers, with evidence of disrupted steroid, sulfur, sugar, bilirubin and bile acid metabolism. Many altered metabolites can be linked to CYP2B6 enzyme, which is known to both metabolize and be modulated by efavirenz[28]. Moreover, the metabolic impact was consistent with other known effects of efavirenz activating pregnane X receptor, constitutive androstane receptor and neuronal receptors[29–31], and its adverse effects on lipid and sugar metabolism[32]. Notably, although increased morbidity and mortality and growth defects were previously reported in HEU infants born to mothers who did not receive efavirenz during pregnancy[6], in our study, the effect of efavirenz on the metabolomic profiles of mothers and infants dominated the differences between HEU and HUU mother-infant pairs. We present here the first MWAS analysis of global effects of efavirenz on human biochemistry. Besides the reported metabolites, many significant metabolite features in the dataset are still unidentified, an area for future investigations. Modern life is in constant chemical exposures, starting in utero. Our study demonstrates that chemical exposures can be tracked and assessed by untargeted metabolomic analysis, adding to the comprehensive understanding of human biochemistry in health and disease.

## Methods

### Cohort enrollment and sample collection

Enrollment took place in at the University of Witwatersrand between June and December 2017. The study was approved by the Ethics Committee of the Witwatersrand University and by the Colorado Multiple Institutions Review Board. The demographic characteristics of the study population, the results of the dietary questionnaire and of the maternal microbiome analysis were previously published[18]. The archived cord and maternal plasma samples collected at delivery from 123 mothers with HIV and 117 mothers without HIV are used here for metabolomics analysis.

### Metabolite and lipid extraction

Plasma samples were maintained on ice throughout the process. For blank, quality control (Qstd), and Standard Reference Material, 20 μL of water, human plasma (MilliporeSigma, cat. no. H4522), or SRM 1950 were aliquoted into labeled tubes, respectively. A pooled quality control (Pool_QC) sample was created by combining 10 μL aliquots from all samples in Batch 1. For extraction, 20 μL of each plasma sample was combined with 100 μL of ice-cold methanol and 4 μL of IS solution, followed by vortexing for 30 seconds and incubation on ice for 10 minutes. Samples were centrifuged at 21,000 *g* at 4°C for 20 minutes, and 80 μL of the supernatant was transferred to autosampler vials for LC-MS analysis. To prepare the internal standard (IS) solution, individual stock solutions of labeled metabolites were prepared in water: 2 mM L-Tyrosine-15N, 10 mM Uracil-15N2, 20 mM L-Glutamic acid-13C5, 5 mM Caffeine-3-methyl-13C, 10 mM L-Methionine-13C5,15N, and 1 M D-Glucose-13C6. A final IS solution was prepared by combining precise volumes of each stock solution with water to achieve desired concentrations, followed by vortexing for 30 seconds.

Lipid extraction involved preparation of an extraction buffer (30 mL MTBE with 1 mL LIPIDOMIX internal standard) and a lipid reconstitution buffer (8 mL MeOH with 1 mL toluene), both maintained at 4°C. Plasma samples were similarly kept on ice. Blank, Qstd, NIST, and Pool_QC samples were prepared as described for metabolomics. For extraction, 20 μL of plasma was mixed with 100 μL of ice-cold methanol and vortexed for 30 seconds. Subsequently, 300 μL of extraction buffer was added, followed by vortexing and addition of 100 μL water for phase separation. After a 10-minute ice incubation, samples were centrifuged at 21,000 *g* at 4°C for 20 minutes. The upper phase (250 μL) was transferred to a fresh tube, dried in a vacuum concentrator at 30°C, and reconstituted in 80 μL of lipid reconstitution buffer. Samples were vortexed, centrifuged again, and 70 μL of the supernatant was transferred to autosampler vials for LC-MS analysis.

### LC-MS and MS/MS Metabolomics analysis

Metabolomics and lipidomics analyses were performed using HILIC and RP C18 columns, respectively, on a Thermo Scientific Orbitrap mass spectrometer, as described previously[33, 34]. The HILIC method utilized an Accucore^™^ 150 Amide column (10×2.1 mm guard; 100×2.1 mm analytical) with 10 mM ammonium acetate mobile phases (95:5 acetonitrile:water, v/v) containing 0.1% acetic acid, with a gradient from 0% to 98% B2 over 20 minutes at a flow rate of 0.55 mL/min and 45 °C column temperature. Lipidomics employed Hypersil GOLD^™^ C18 columns (10×2.1 mm guard; 50×2.1 mm analytical) with 10 mM ammonium formate mobile phases (60:40 acetonitrile:water for A1; 90:10 isopropanol:acetonitrile for B1) containing 0.1% formic acid, using a gradient from 15% to 99% B1 over 20 minutes at a flow rate of 0.4 mL/min and 45 °C. MS data were acquired in positive and negative ionization modes (66.7–1000.0 m/z, resolution 60,000 at m/z 200) with optimized ion source parameters, including 3.5 kV/2.5 kV spray voltage, 300 °C capillary temperature, and 425 °C heater temperature.

Data dependent MS2 (DDA) was applied to subpool samples over mass ranges (66.7–1000.0 m/z) using the following settings: for Full MS, resolution = 60,000; AGC target = 3e6; Maximum IT = 50 ms; for dd-MS^2^: resolution = 30,000; AGC target = 1e5; Maximum IT = 50 ms; Isolation width = 1.0 m/z; stepped normalised collision energies = 20, 30, 40%.

### Data acquisition and preprocessing, annotation

Metabolomics data from four acquisition modes (HILIC ESI+, HILIC ESI−, RP ESI+, RP ESI−) were processed using asari version 1.14[33] and the PCPFM pipeline version 1.1[35]. Processed feature tables were blank-masked, outlier-filtered, normalized by TIC, log2 transformed, and batch-corrected before downstream statistical analyses. Batch-corrected feature tables were imported into Python (v3.11) using pandas. Sample metadata were merged to create comprehensive sample–feature mappings, including participant status (HEU vs. HUU) and sample type (maternal or cord plasma). Annotations at multiple confidence levels (MS/MS-based L2–L1a) were indexed for downstream analysis.

### Statistical analysis

For univariate analysis, Welch’s t-tests were applied to log-transformed feature intensities for HEU vs. HUU groups in both maternal and cord samples to account for heteroscedasticity. Multiple-testing correction was applied using Bonferroni adjustment for high specificity, while false discovery rate (FDR, Benjamini–Hochberg) was computed for complementary interpretation. Correlation networks between drug-related features and other metabolites were computed using pairwise Pearson correlations within group-specific subsets (HEU or HUU). Observed correlation distributions were compared to null distributions generated through permutation testing (1,000 iterations), yielding empirical z-scores and permutation-based p-values to assess enrichment beyond random expectation. Significant associations were visualized in enrichment histograms with annotated overlays.

### Data visualization

Volcano plots were generated using matplotlib to visualize statistical significance (−log_10_ p-values) versus effect size (log_2_ fold change). Points were color-coded by significance category: both fold-change and p-value significant, p-only, fold-change-only, or non-significant. Annotations for prioritized metabolites (Levels 1a–2) were automatically applied to significant features. Plots were generated separately for maternal and cord plasma. For metabolites of particular interest (e.g., drug-related compounds and key metabolic markers), boxplots were generated to display group-wise distributions. These plots utilized alpha-transparency overlays to indicate replicate density and included adjusted p-values (raw, Bonferroni, and FDR) to support feature-level interpretation.

For multivariate visualization, data were z-score standardized and subjected to PCA using scikit-learn. Plots of the first two principal components were generated, stratified by HIV exposure status and maternal vs. cord designation. 95% confidence ellipses were overlaid based on the covariance structure of each group to highlight clustering.

Plots were generated with matplotlib and adjustText for clear annotation and high-resolution output. Colors were standardized (HEU: #D41159, red; HUU: #1A85FF, blue) across all plots for consistency. Boxplots, PCA, volcano, and enrichment plots were archived to a reproducible directory structure for integration with manuscript figures. All analyses were scripted in Python (pandas, numpy, scikit-learn, matplotlib, scipy), and all notebooks were version-controlled to ensure reproducibility.

### Pathway and Enrichment Analysis

Significant features were formatted for pathway enrichment analysis using Mummichog version 2.7. Separate input tables were generated for maternal and cord samples per ionization mode, including m/z, retention time, raw p-value, and test statistic. Mummichog was executed to identify pathways disproportionately associated with significant metabolites, and pathway tables were sorted by overlap size and adjusted significance for downstream visualization.

## Supplementary Material

Supplement 1

Supplemental Figure 1. Overview of HILIC-metabolomics data.

a) PCA plot and b) clustermap showing the separation of HEU/HUU and maternal/cord samples in HILIC-metabolomics data.

Supplemental Figure 2. Additional significant metabolites based on HIV exposure status, both infants and mothers.

**Supplemental Figure 3. Many significant differential metabolites are mapped to a steroid metabolism network.** All shown metabolites are significant in the mothers’ data. Red indicates higher abundance in the HEU group.

a) Network of altered steroids due to HIV exposure in mothers.

b) Same network in a) but colored by fold change in the infants’ data.

Supplemental Figure 4. Additional result on efavirenz exposure.

a) Correlation plot highlighting the positive correlation of 8-hydroxyefavirenz between paired maternal and cord plasma samples.

b) Manhattan plot comparing the distribution of features correlated with Efavirenz versus features that are significantly different due to HIV exposure in maternal plasma samples along the retention-time axis.

c) Dot plot summarizing the pathway analysis results of efavirenz and HIV exposure status associated features in maternal and cord samples in RP-data, which have limited pathway coverage.

## Figures and Tables

**Figure 1.**
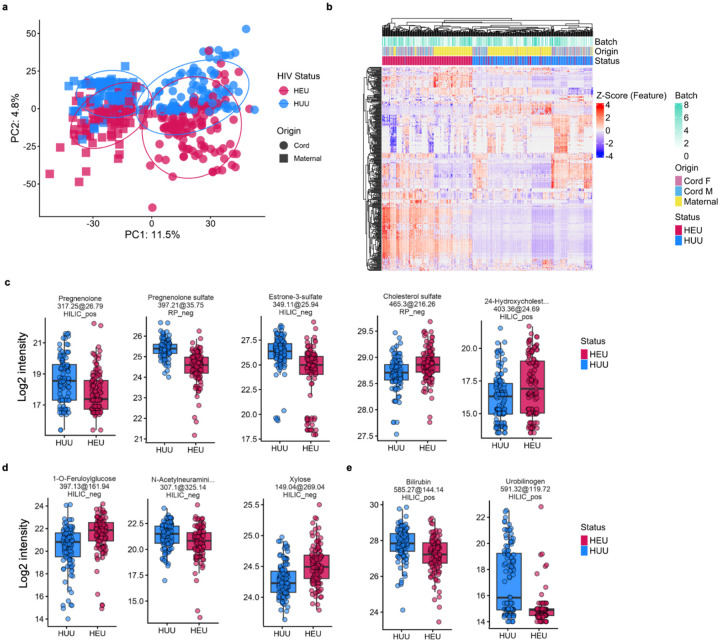
Distinct biochemical phenotypes of mother-infant dyads by metabolomic profiling. a) PCA plot showing the separation of HEU/HUU and maternal/cord plasma samples in metabolomics, using all features in RP- (negative ionization from reverse phase chromatography). b) Cluster heatmap of metabolomic features (q<0.05, RP-), samples largely cluster by HIV exposure status. c-e) Boxplots of example significant metabolites.

**Figure 2.**
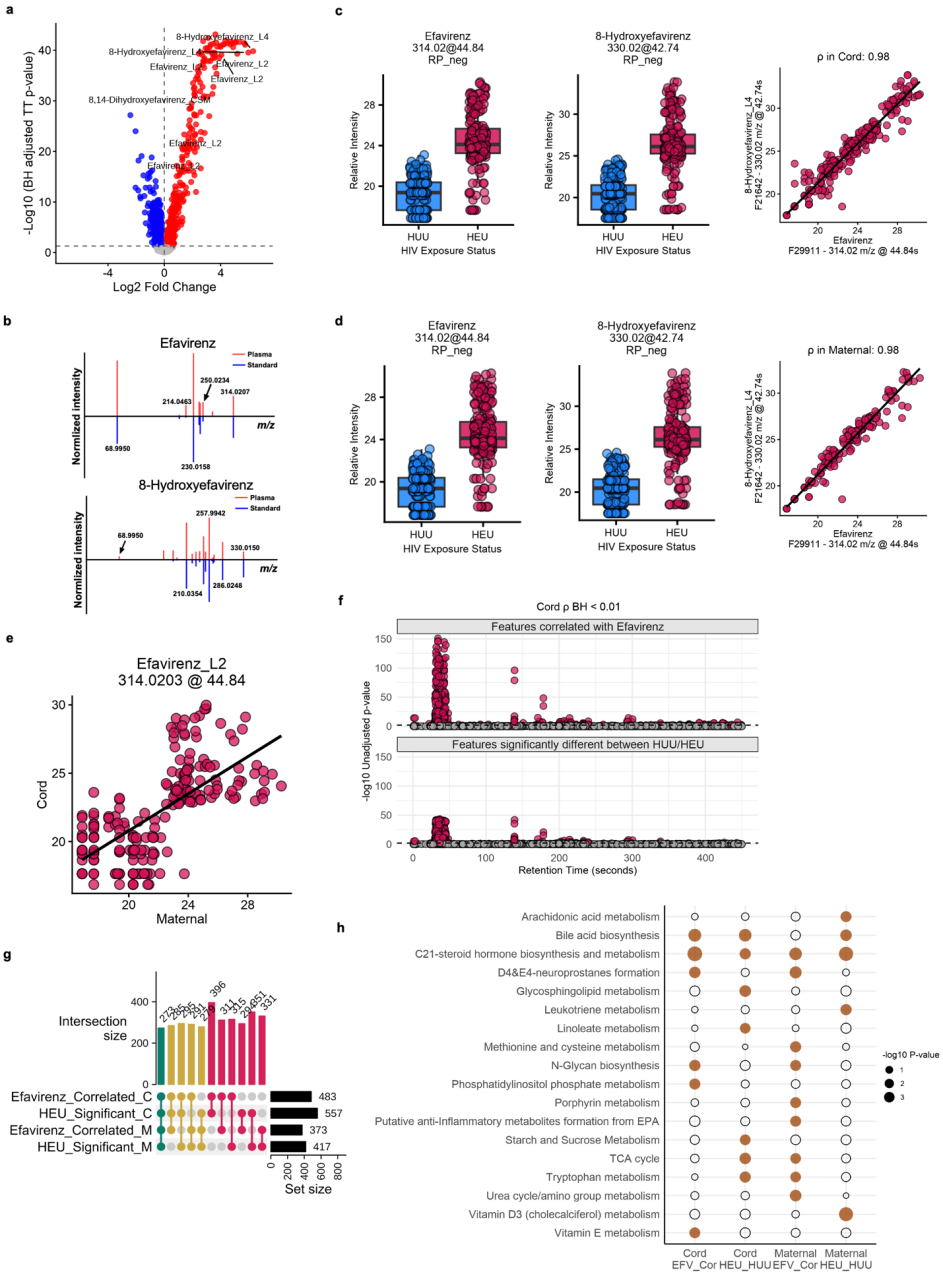
Efavirenz drives differential metabolites in HEU infants. a) Volcano plot of metabolomic features significantly different between HEU and HUU infants (RP-, Efavirenz and its metabolites are labeled). b) MS/MS identification of efavirenz and 8-hydroxyefavirenz. c) Detection of efavirenz and 8-hydroxyefavirenz in infants (RP-); each dot in boxplot represents a subject. The baselines were not zero because the compounds have residuals in chromatographic columns. d) Detection of efavirenz and 8-hydroxyefavirenz in mothers (RP-); each dot in boxplot represents a subject. e) Correlation of efavirenz levels between infants and their mothers. f) Manhattan plot comparing the distribution of features correlated with Efavirenz versus features that are significantly different due to HIV exposure in cord plasma samples along the retention-time axis (RP-). g) UpSet plot showing the intersection of features significantly correlated with efavirenz and features significantly associated with HIV exposure status (RP-). h) Significant pathways overlap between efavirenz correlates and HEU/HUU differences (HILIC+). The HILIC+ data have more pathway coverage compared to RP- data (Suppl Fig. 4c).
