# Supplementary material for: Distinct biochemical phenotypes of HIV exposed infants driven by antiviral medication
Source: medRxiv. 2026 Feb 2:2026.01.28.26344948. Preprint. [Version 1] doi: 10.64898/2026.01.28.26344948 (PMC12889808; doi:10.64898/2026.01.28.26344948)
Supplement: Supplement 1 [file media-1.pdf]

a b

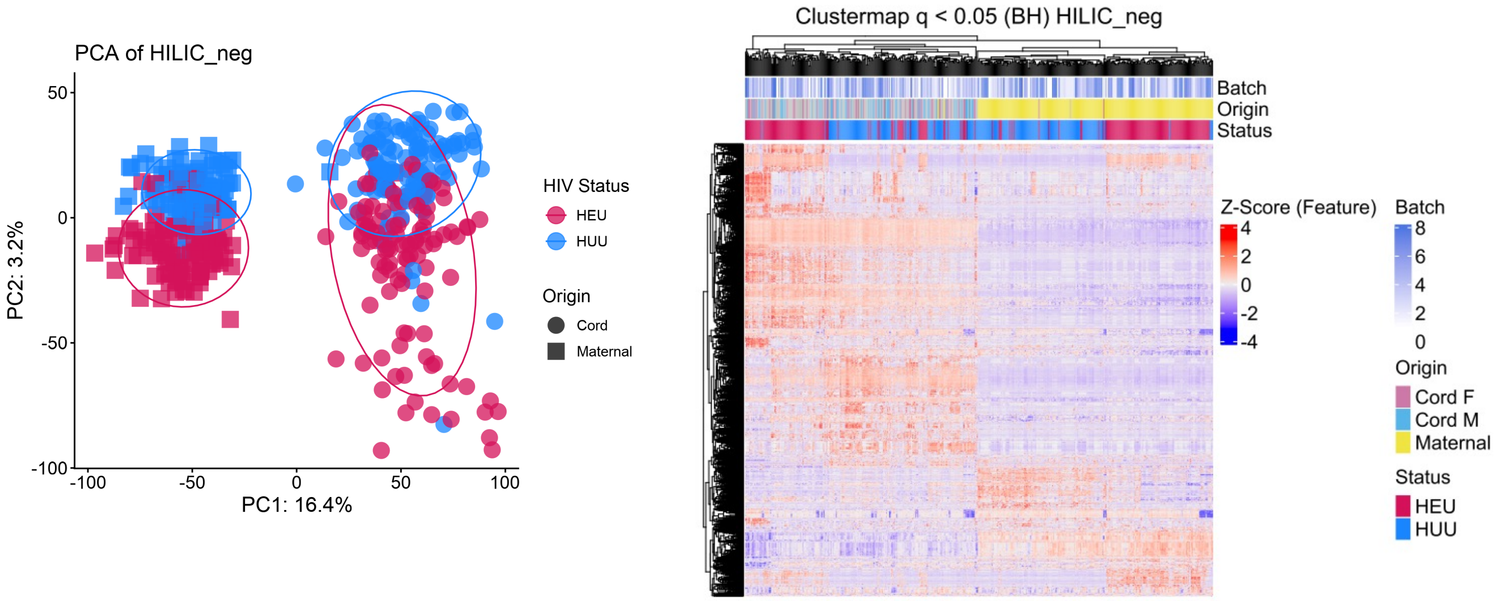

Supplementary Figure 1

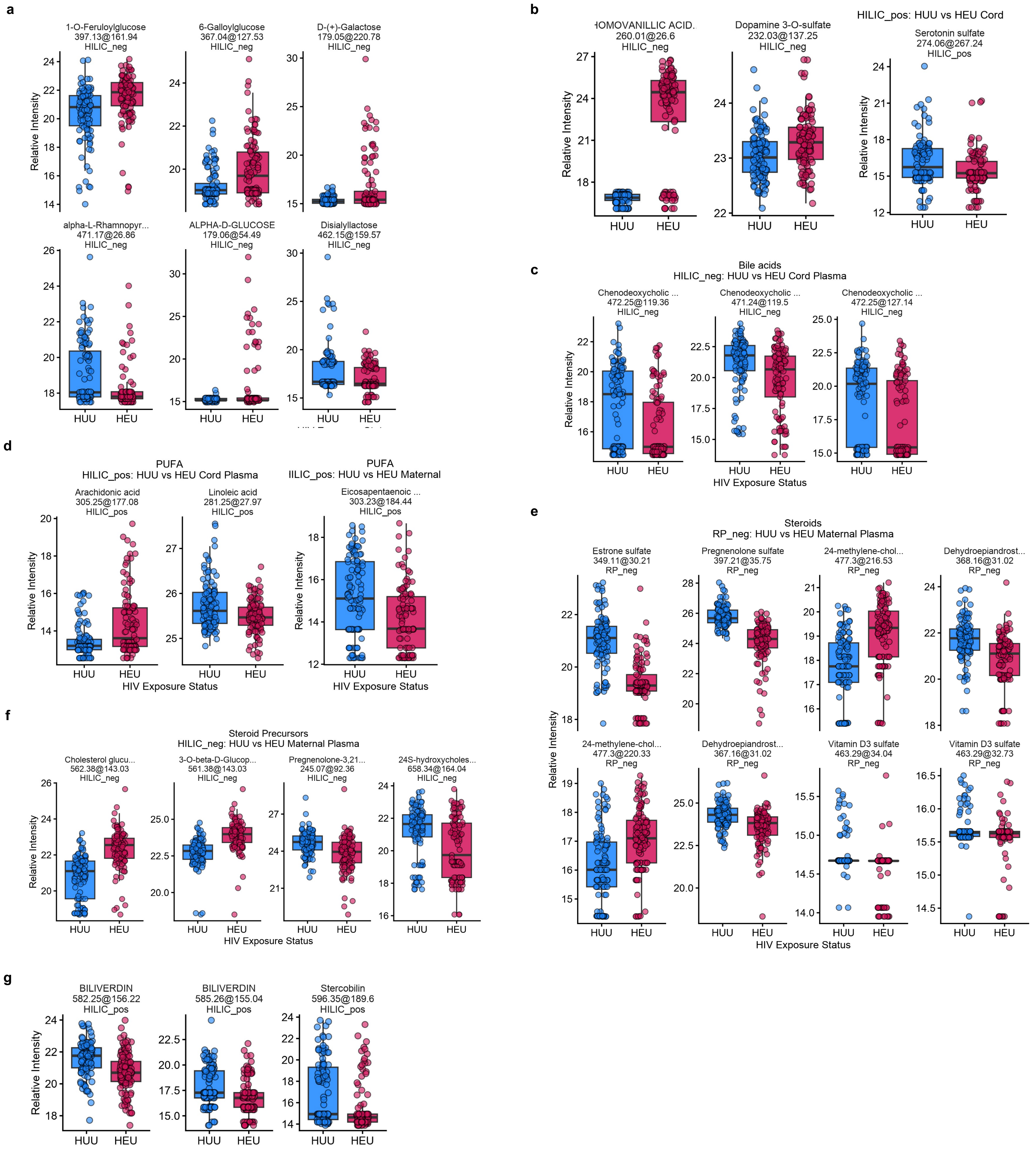

Supplementary Figure 2

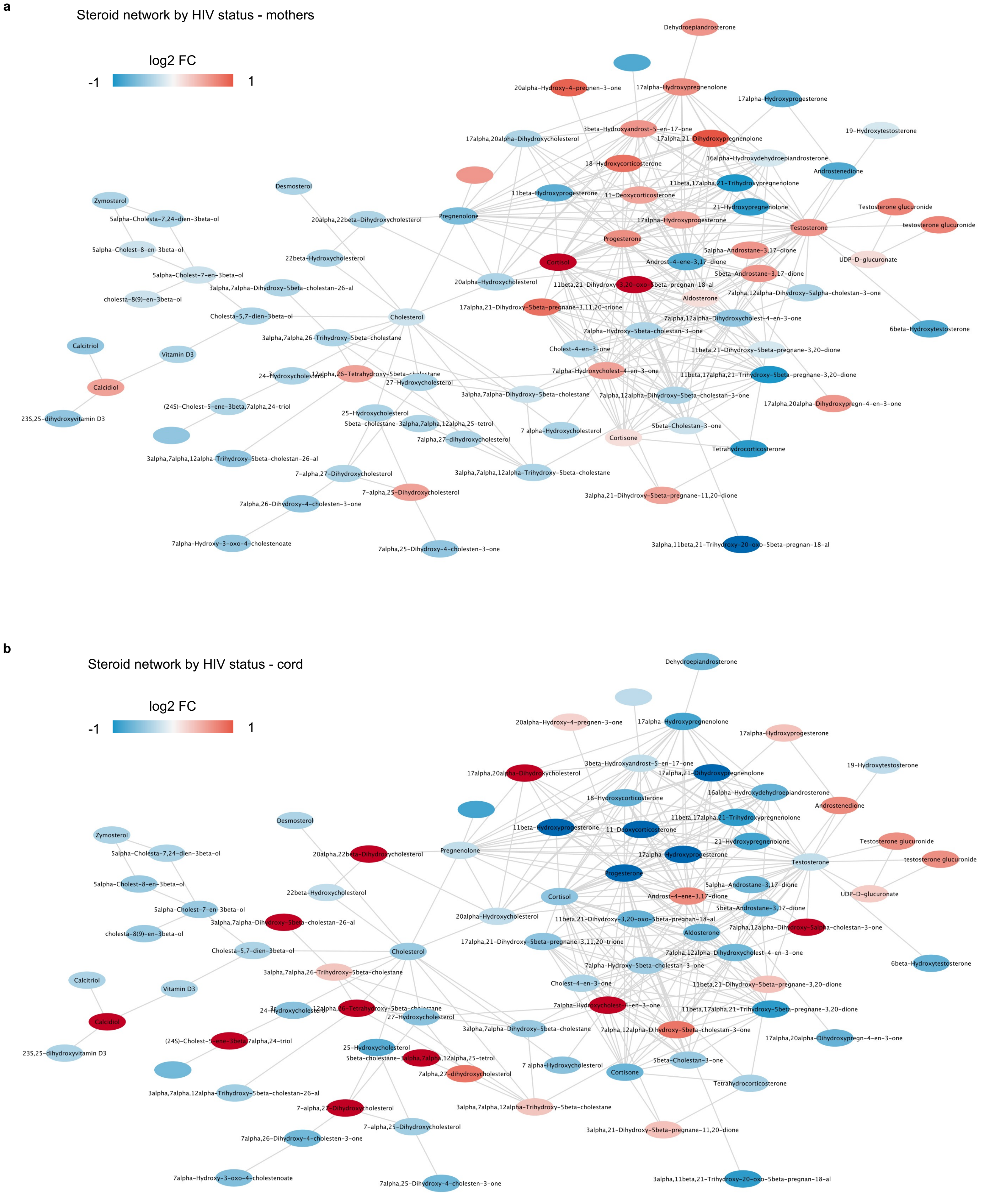

Supplementary Figure 3

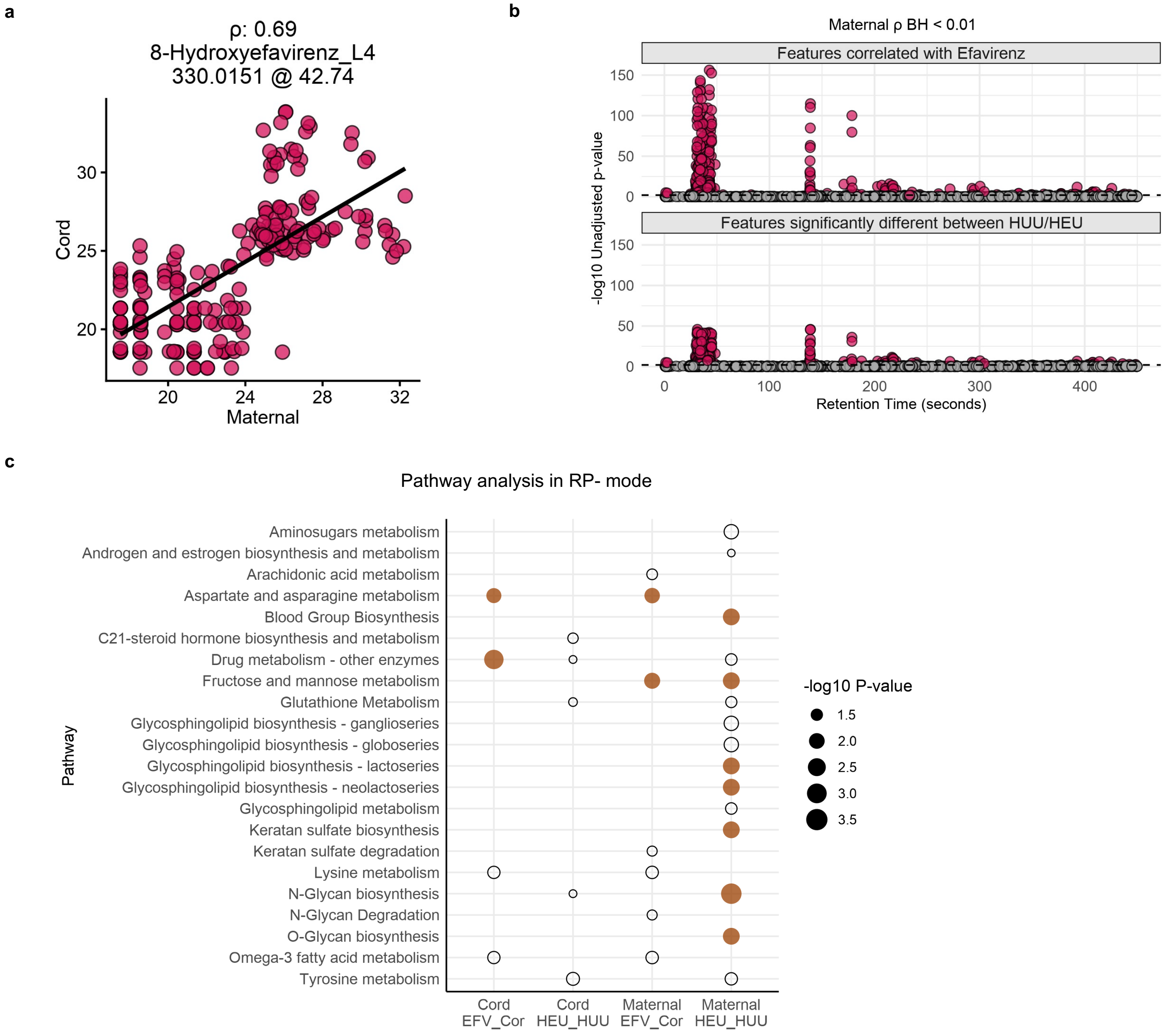

Supplementary Figure 4
